# Establishment of Rapid Detection Methods for rs76971248 Related to Leukemia

**DOI:** 10.1155/2022/9847708

**Published:** 2022-03-29

**Authors:** Li-Yan Sun, Zhen Li, Song-Xing Wang, Hao Chen, Ze-Tao Sun, Long Peng, Liu-Mei He, Yun-Ping Xu

**Affiliations:** ^1^Institution of Transfusion Medicine, Shenzhen Blood Center, Shenzhen, Guangdong 518035, China; ^2^Department of Transfusion Medicine, School of Laboratory Medicine and Biotechnology, Southern Medical University, Guangzhou, Guangdong 510515, China

## Abstract

**Background:**

The HLA-E gene is a member of the HLA-I gene family. Its genetic polymorphism is regarded as associated with numerous diseases. Establishing a rapid and accurate detection method of disease-related SNP sites in HLA-E is particularly important.

**Methods:**

Blood samples from 226 healthy blood donors and 228 leukemia patients were collected, and DNA was extracted. Three typing methods based on PCR-sequence-based typing, TaqMan genotyping, and high-resolution melting curve were established to identify rs76971248 (G>T). The Chi-square test was used for statistical analysis by SPSS.

**Results:**

Three methods based on PCR-SBT, TaqMan genotyping, and HRM were all able to identify rs76971248. The software for analyzing the results of HLA-E sequencing was easy to use, and the results were accurate. The frequency of rs76971248 in different types of leukemia patients was significantly lower than that in healthy blood donors (*p* < 0.05). And the frequency of the G/G genotype in leukemia patients was significantly higher than that in healthy blood donors (*p* < 0.05).

**Conclusions:**

For the screening of known SNP sites in large-scale populations, among the three methods, the TaqMan genotyping method had the advantage of shortest time consumption, simplest operation, and greatest specificity, which was the most appropriate method for this experiment. The analysis software for HLA-E gene sequencing needed to be further optimized. rs76971248 had a protective effect against leukemia. And the G/G genotype was a risk factor for leukemia.

## 1. Introduction

HLA genes are on the short arm of chromosome 6 at the 6p21 locus, which is divided into three groups based on gene sequence similarities and functions, classes I–III. The HLA class I gene contains classical (HLA-A, HLA-B, and HLA-C) and nonclassical HLA class I genes (HLA-G, HLA-E, and HLA-F). The HLA-E gene is located between HLA-A and HLA-C. It is about 4 kb in length, including 8 exons and 7 introns. Currently, the HLA-E gene is actively studying, and the number of HLA-E alleles has increased up to 256 HLA-E alleles, which encode 110 HLA-E proteins [1]. The HLA-E molecule encoded by the HLA-E gene is a ligand for the surface receptors of NK cells and T cells, which can affect the body's immune response [2–4]. Under certain pathological conditions, especially for tumors, high expression of HLA-E molecules on the surface of tumor cells is considered to inhibit the body's immune response [5].

As we all know, different SNP sites have different effects on molecule expression. Many researchers focus on SNP sites in the exon region which can alter amino acids, thereby changing the expression of the molecule [6]. In recent years, it has been proved that most disease-related SNP sites are located in the regulatory region [7–9]. Polymorphic sites in regulatory regions may affect gene expression levels through different mechanisms (such as differential binding of transcription factors or microRNA binding) [10]. However, there are limited studies on the association of SNP sites in HLA-E regulatory regions and diseases.

The team behind this article has been engaged in the typing and analysis of the HLA-E gene for many years and established the gene full-length amplification and sequencing typing method. We first reported the full-length sequence of HLA-E∗01:01:01:06, containing one SNP site in HLA-E regulatory regions (rs76971248 NT-26G>T) [11]. Moreover, we also found a correlation between HLA-E gene polymorphism and leukemia [12, 13], especially for HLA-E∗01:01:01:06. Therefore, in this article, we first reported the relationship between rs76971248 and different types of leukemia. And we first established three methods (PCR-SBT, TaqMan genotyping method, and HRM) to detect rs76971248 and found the most suitable method for screening rs76971248 in the large-scale population.

## 2. Methods

226 healthy blood donors were selected as research subjects in the Shenzhen Blood Center from January to September 2020 (Supplement Figure). Criteria for inclusion in this experiment were as follows: ① age in the range of 18-60 years old, ② results of predonation physical examination and related hematological examinations meet the requirements of the Blood Donor Health Examination (GB18467-2011), and ③ the subjects follow the relevant requirements in the Shenzhen Special Economic Zone Regulations on Unpaid Blood Donation for specified healthy, voluntary, and unpaid blood donors. Exclusion criteria were as follows: ① blood donors with a family history or previous history of major diseases and ② involuntary participation in the trial.

228 leukemia patients were selected as research subjects in the Shenzhen Blood Center from January to September 2020. Criteria for inclusion in this experiment were as follows: ① patients with stable leukemia and ② patients who had not undergone a transplant. The exclusion criteria were as follows: ① patients with major diseases of other systems and ② involuntary participation in the trial.

This study was approved by the Medical Ethics Committee of Shenzhen Blood Center (No.: SZBC2019R021). All participants signed an informed consent form.

### 2.1. PCR-Sequence-Based Typing Method

Whole blood (5 ml) from each subject was placed in a dipotassium ethylenediaminetetraacetate anticoagulation tube and mixed well, and 400 *μ*L was taken to extract 150 *μ*L of DNA with a purity of 1.8-2.0. The extracted DNA concentration of each sample was adjusted to 50-100 ng/*μ*l, according to the full sequence of HLA-E gene published in the IPD-IMGT/HLA database. The Oligo 6.0 software was used to design HLA-E gene promoter region amplification and sequencing primers. The primer sequences used for PCR amplification are as follows: forward primer, 5′-GCCCAGCCAGGACTAATTTCT-3′ and reverse primer, 5′-TCTCTACTCCCGGTAGAGGCC-3′. The PCR reaction system included 2× Pro Taq Master Mix: 12.5 *μ*L, DNA: 4 *μ*L, Primer Mix: 1 *μ*L, and RNAse-free water, 7.5 *μ*L. The PCR reaction conditions were as follows: 94°C for 30 s, 1 cycle; 98°C for 30 s, 60°C for 30 s, and 72°C for 1 min, 35 cycles; and 72°C for 2 min, 1 cycle. The length of the amplified product was approximately 400 bp.

Enzymes were used to purify the amplified product, and the purification system was as follows: PCR amplified product, 10 *μ*L; exonuclease I, 0.5 *μ*L; shrimp alkaline phosphatase, 1 *μ*L; and RNAse-free water, 0.5 *μ*L. The purification procedure was as follows: 37°C for 30 min and 80°C for 20 min, and the purified product was sequenced with a sequencer. The sequencing reaction system consisted of the following: BigDye® Terminator v3. 1 Ready Reaction Premix, 0.4 *μ*L; 5x sequencing buffer, 1.8 *μ*L; sequencing primer, 1 *μ*L; RNAse-free water, 5.8 *μ*L; and PCR purified product, 1 *μ*L. All the above operations were carried out in strict accordance with the reagent and instrument instructions.

### 2.2. TaqMan Genotyping Method

The Oligo 6.0 software was used to design PCR amplification primers and probes of the rs76971248. The system of TaqMan genotyping method consisted of the following: forward primer, 0.5 *μ*L; reverse primer, 0.5 *μ*L; wild-type probe, 0.5 *μ*L; mutant probe, 0.5 *μ*L; LightCycler 480 Probe Master, 10 *μ*L; DNA, 5 *μ*L; and RNase-free water to make up the volume to 20 *μ*L. The amplification program of the TaqMan genotyping method was set as follows: 95°C for 10 min, 1 cycle; 95°C for 10 s, 60°C for 1 min, and fluorescence signal collection at 72°C for 1 s, 40 cycles; and 40°C for 30 s.

### 2.3. High-Resolution Melting Curve Method

The Oligo 6.0 software was used to design high-resolution melting curve amplification primers for HLA-E gene regulatory region fragments containing rs76971248, and the DNA concentration of all specimens was adjusted to 10 ng/*μ*l. The reaction system for high-resolution melting curve amplification is as follows: 2x HRM PCR Master Mix, 12.5 *μ*L; DNA, 5 *μ*L; 10 *μ*M Primer Mix, 1.75 *μ*L; and RNAse-free water, 5.75 *μ*L. The program of the high-resolution dissolution curve method was as follows: 95°C for 3 min, 1 cycle; 95°C for 10 s, 51°C for 10 s, and 72°C for 20 s (signal acquisition in this step), 50 cycles; 95°C for 1 min, 40°C for 1 min, then the temperature was raised from 60°C to 95°C, and the signal was continuously collected to create a dissolution curve, 40°C for 30 s, 1 cycle.

### 2.4. Statistical Analysis

The analysis software we designed for HLA-E gene sequencing was used to analyze the sequencing results of PCR-SBT. The endpoint genotyping in the program of Roche LightCycler 480 was used to analyze the results of the TaqMan genotyping method, and the gene scanning in the program of Roche LightCycler 480 was used to analyze the results of the HRM method. All statistical analyses were performed using the SPSS software (https://www.ibm.com/analytics/spss-statistics-software).

## 3. Results

### 3.1. The PCR-SBT Method Was the Most Accurate Method among the Three Methods

**Figure 1:**
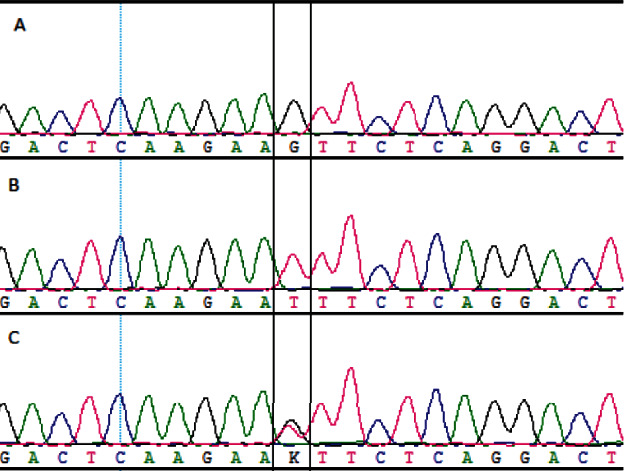
The sequencing peak maps of G/G, T/T, and G/T genotypes.

**Figure 2:**
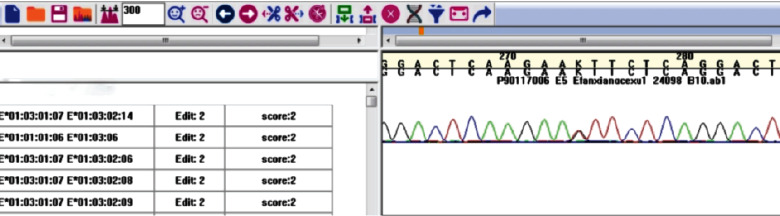
The software results of the G/T genotype.

The PCR-SBT method was used to detect rs76971248 (G>T). The results of the genotype were G/G (Figure 1(a)), G/T (Figure 1(c)), and T/T (Figure 1(b)). The sequence of the HLA-E gene was analyzed by the software we designed. For example, when the genotype was G/T, the software results of the partial sequencing are shown in Figure 2. The combination of alleles with the highest score was G/T base heterozygous in that position; hence, the genotype of this sample was G/T. All samples with a concentration that met the requirements can be accurately typed. Excluding human operation errors, the genotypes of all samples can be typed with this method. The results of our software were consistent with the results of Clustal X.

In Supplement Table 1, the results of the SPSS software showed that the hypothesis of Hardy–Weinberg equilibrium in healthy blood donors and leukemia patients was accepted (*p* > 0.05). In Supplement Tables 2 and 3, there was no significant difference between with age or gender among healthy blood donors (*p* > 0.05).

As shown in Table 1, the greatest statistical difference between the two groups, 226 healthy blood donors and 228 leukemia patients, was still observed in the frequency of rs76971248 (G>T) (9.29% of healthy donors vs. 4.16% of leukemia patients, *p* = 0.002). Whether in patients with lymphocytic leukemia or myeloid leukemia, the frequency of rs76971248 (G>T) was lower than that of healthy blood donors in Table 2 (*p* < 0.05).

In Table 3, the frequency of the G/G genotype in leukemia patients was higher than that in healthy blood donors (81.86% of healthy donors vs. 92.11% of leukemia patients, *p* < 0.05). And the frequency of the G/T genotype in leukemia patients was lower than that in healthy blood donors (17.70% of healthy donors vs. 7.89% of leukemia patients, *p* < 0.05).

### 3.2. The TaqMan Genotyping Method Was the Fastest Detection Method among the Three Methods

**Figure 3:**
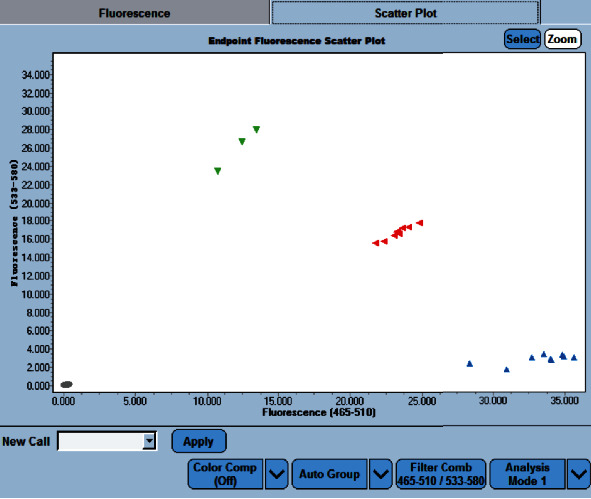
The endpoint genotyping results of T/T, G/T, and G/G genotypes.

The results of the TaqMan genotyping method for rs76971248 were consistent with the results of PCR-SBT method. Allele Y (green) in Figure 3 indicated that the genotype was T/T. Both alleles (red) indicated that the genotype was G/T, and Allele X (blue) indicated G/G. Unknown alleles (pink) indicated that the result for this sample was inaccurate, and it needed to repeat the experiment.

### 3.3. The HRM Method Was the Most Sensitive Method among the Three Methods

**Figure 4:**
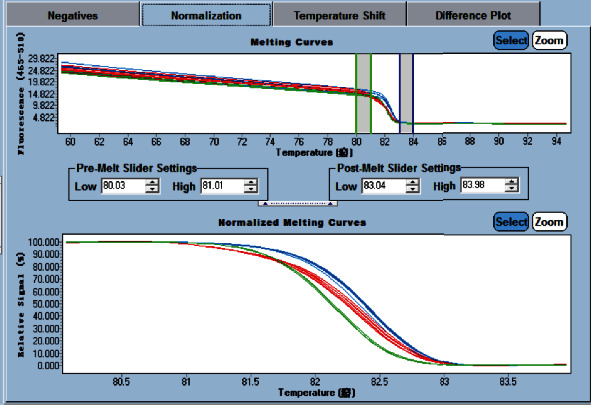
The gene scanning results of T/T, G/T, and G/G genotypes.

The results of the HRM of rs76971248 are shown in Figure 4. Genotype 1 (green) meant G/G, genotype 2 (red) meant G/T, and genotype 3 (blue) meant T/T. The results of rs76971248 using the HRM method were consistent with the results of the TaqMan genotyping method and PCR-SBT. In addition, genotype 4/5/6 (pink/grey/yellow) may mean that there are other mutations in the amplification fragment. Unknown genotype (crimson) may mean that the result for this sample was inaccurate, and it needed to repeat the experiment. All specimens require at least three replicate wells for experiments.

## 4. Discussion

In recent years, the correlation between HLA-E SNP sites and disease has received increasing attention from researchers [14]. Most of them are in the exon region. For example, it was reported that rs1264457 (NT756 G>A) and rs1059510 (NT424 T>C) of HLA-E gene were associated with some inflammatory diseases [15]. While the discovery of polymorphisms in the regulatory region has greatly enriched [16], the relationship of HLA-E SNP sites in the regulatory region and tumors has not been reported, especially for leukemia. However, there is already evidence that the HLA-E∗01:03 allele is an independent predictor of the early treatment needs of CLL patients [17], which proves the polymorphism of the HLA-E gene, as a disease marker, plays an important role in the occurrence and development of leukemia. The reason why there is no relevant research on the SNP sites of the HLA-E regulatory region and leukemia may be the low frequency of these SNP sites in some populations and the lack of suitable methods for screening them.

In this article, we established three methods (PCR-SBT, TaqMan genotyping, and HRM) to detect rs76971248 among healthy blood donors and leukemia patients. And we found that the frequency distribution of rs76971248 was not related to age and gender by analyzing the frequency distribution of rs76971248 in different gender and age groups among healthy blood donors (Supplement Tables 2 and 3, *p* > 0.05). The frequency of rs76971248 was lower in different types of leukemia patients than that in healthy blood donors (*p* < 0.05), which indicated that rs76971248 has a protective effect against leukemia. In addition, the frequency results of different genotypes showed that the G/G genotype was a risk factor for leukemia, and the G/T genotype was a protective factor for leukemia. Because of the small number of individuals with the T/T genotype, we could not draw conclusions about the role of this genotype in leukemia.

HLA-E molecules are ligands for NK cell surface receptors. Its high expression is thought to suppress the immune response of NK cells. It has been proved that rs76971248 reduced the transcriptional activity of the promoter region of HLA-E. Therefore, we suspect that rs76971248 is very likely to be related to different types of leukemia because it has lower surface expression, thereby resulting in its lower inhibitory effect on cytotoxic lymphocytes, just like some HLA-E alleles. So, leukemia patients with T/T or G/T genotypes are more conducive for immune cells killing leukemia cells compared with those with G/G genotype. NK cell immunotherapy has been widely used in the treatment of various types of leukemia [18]. Before NK cell infusion, it is necessary to evaluate its treatment sensitivity. Leukemia cells with high expression of HLA-E molecules are more likely to escape the immune response of NK cells, and it is not conducive to the application of NK cell-related therapies. Leukemia patients who are typed as T/T or G/T genotype with lower HLA-E molecule expression of leukemia cells may have a better therapeutic effect of NK cell infusion compared to the patients with G/G genotype.

In previous studies, many methods were used to partially or full-length sequence the HLA-E gene containing some SNP sites, including PCR-SBT and PCR-SSP [19, 20]. Among these, the PCR-SBT method is considered as the gold standard and its results are accurate and comprehensive; it can help discover new SNP sites. But its experimental process is time-consuming and complex, which is no longer efficient for research on the association of known SNP sites with diseases among different populations. And the results of the sequence must be analyzed by various software programs. The analysis processes are also complicated and time-consuming. As a result, the research team developed the analytical software for the HLA-E gene sequence, which included the HLA-E gene allele sequences in the IPD-IMGT/HLA database. After importing the full-length or segmented sequencing peak map of the HLA-E gene, the software can analyze the typing results. The results of this software are very accurate, especially for the full-length sequencing of the HLA-E gene, which saves time for analysis, but the details are still not perfect. It has strict requirements in the name of imported sequence files; hence, it still needs to be further optimized. In this article, even if this software is available to help save time, the PCR-SBT method still takes the longest time for large-scale screening of rs76971248 compared to the HRM method and the TaqMan genotyping method. But if the purpose is to discover the new alleles or explore the full-length gene polymorphism, the PCR-SBT method is necessary and convenient, and it is the best choice compared to the other two methods.

With the development of quantitative fluorescence techniques, there are many methods to detect the SNP sites. In recent years, the TaqMan genotyping method and the HRM method have become more popular.

The TaqMan fluorescent probe is an oligonucleotide probe, and its 5′ end carries a fluorescent group, such as FAM, TET, VIC, and HEX, and the 3′ end carries a quenching group, such as TAMRA and BHQ, and every time a DNA strand is amplified, a fluorescent molecule is formed. The accumulation of fluorescent signals is fully synchronized with the formation of PCR products. Therefore, the TaqMan genotyping method has the advantages of high specificity, good reproducibility, short time consumption, and simple operation. It is widely used in the detection of SNP sites of various genes, including some SNP sites in the HLA-E gene [21, 22]. It can quickly and accurately identify the target SNP sites among different populations but also has limitations. One is that the probes and primers used in this method can only be designed for known SNP sites, and it is impossible to identify whether the amplified fragment contains other mutations. The other is that when the DNA concentration of the samples is low, it may affect the judgment of the result. In this article, the TaqMan genotyping method was first established to detect rs76971248. The advantages we mentioned above of this method had been verified in this experiment. It was the most time-saving detection method with the simplest operation of rs76971248 genotyping among the three methods. The results of samples with a good DNA concentration were all accurate. So, when the concentration of DNA is over 50 ng/*μ*l and the purpose of the experiment is to screen known SNP sites among large-scale populations, the TaqMan genotyping method is the best choice compared to the other two methods.

The HRM method is that the thermal stability of double-stranded nucleotides is affected by their length and base composition, and sequence changes will cause changes in the melting of double-stranded nucleotides during the heating process. The fluorescent dyes used can only be embedded and bound to double-stranded nucleotides. Therefore, real-time PCR technology can be used to detect the changes in the fluorescence signal during the melting of double-stranded nucleotides in a way that generates melting curves of different shapes. The differences in the PCR products are visually displayed. Different SNP sites can be distinguished through the corresponding analysis software. The HRM method can find the differences of a single base in the amplified fragment, so it can help discover the unknown SNP sites, because it is high-throughput, low cost, does not require the use of sequence-specific probes, and has extremely high sensitivity. As a result, this method has been widely used to detect SNP sites and unknown mutations [23, 24]. However, there are many limitations of this method compared to the other two methods. It may cause deviations in the judgment of target mutations. For example, if there are other SNP sites due to reasons such as blood freezing for a long time in the amplified fragments, or if the initial concentration of samples is not consistent, it can lead to different results. In addition, this method requires higher temperature sensitivity of the instrument. In this article, the HRM method was first established to detect rs76971248, but the accuracy of the results of the HRM method was not as good as the PCR-SBT method; sometimes its results needed to be repeated multiple times to avoid inaccurate genotyping caused by human manipulation. Its operation was more complicated, and the experimental process took longer compared to the TaqMan genotyping method. This method is not suitable for large-scale population screening experiments with known SNP sites. But if the concentration of DNA is not high, due to reasons such as blood freezing for a long time, this method can get the results. If the purpose is to discover unknown mutations in one short fragment, this method is a good choice, because the HRM method takes less time and does an easier job compared to the PCR-SBT method.

In summary, the lower frequency of rs76971248 in different types of leukemia patients may indicate that rs76971248 is not conducive to the occurrence and development of two types of leukemia. The results of the three methods used for the detection of rs76971248 are the same. Each method has its advantages and limitations. For different purposes, we should choose a suitable method to experiment. If the purpose of the experiment is to confirm the sequence of the new allele, the PCR-SBT is the best choice. If the purpose is to screen for the known SNP sites among a large-scale population, the TaqMan genotyping method was the best choice. If the purpose is to find some mutations in one short fragment, or the DNA concentration of samples is low, the HRM method is the best choice. In this article, in order to study the frequency distribution of rs76971248 among healthy people and patients with different types of leukemia, among the three methods, the TaqMan genotyping method is the most suitable for this experiment.

## 5. Conclusion

rs76971248 has a protective effect against leukemia. And the G/G genotype is a risk factor for leukemia. Among the three methods, the TaqMan genotyping method is the best choice for screening rs76971248 in large-scale populations. The HLA-E sequence analysis software can analyze both the full-length and partial sequencing results to draw conclusions.

## Figures and Tables

**Table 1:** The frequency of rs76971248 between healthy blood donors and leukemia patients.

Group	Frequency		*p* value	*χ* ^2^	OR	95% CI
	NT-26G	NT-26T				
Leukemia patients	437 (95.84%)	19 (4.16%)	0.002	9.515	0.424	0.243-0.742
Healthy blood donors	410 (90.71%)	42 (9.29%)				

**Table 2:** The frequency distribution of rs76971248 among different types of leukemia patients.

Leukemia group	Number^∗^	NT-26T	NT-26G	*p* value	*χ* ^2^
Lymphocytic leukemia	56	4 (3.57%)	108 (96.43%)	0.048	3.921
Myeloid leukemia	154	14 (4.54%)	294 (95.45%)	0.014	6.046

^∗^18 patients with unclear leukemia classification.

**Table 3:** The frequency of three genotype between healthy blood donors and leukemia patients.

Group	Frequency		
	G/G	G/T	T/T
Leukemia patients	210 (92.11%)	18 (7.89%)	0
Healthy blood donors	185 (81.86%)	40 (17.70%)	1 (0.44%)
*p* value	0.019	0.002	0.498^∗^
OR	2.007	0.399	
95% CI	1.114-3.614	0.221-0.719	

^∗^Fisher's precision probability test was performed.

## Data Availability

All data generated or analyzed during this study are included in this published article and its supplementary information files.

## Supplementary Materials

Supplementary Materials Supplement Table 1: Hardy–Weinberg equilibrium test. Supplement Table 2: correction between HLA-E gene nucleotide-26 with genUPder among healthy blood donors. Supplement Table 3: correction between HLA-E gene nucleotide-26 with age among healthy blood donors.
