# Supplementary material for: Establishment of Rapid Detection Methods for rs76971248 Related to Leukemia
Source: Dis Markers. 2022 Mar 29;2022:9847708. doi: 10.1155/2022/9847708 (PMC8983173; doi:10.1155/2022/9847708)
Supplement: Supplementary Materials — Supplement Table 1: Hardy–Weinberg equilibrium test. Supplement Table 2: correction between HLA-E gene nucleotide-26 with genUPder among healthy blood donors. Supplement Table 3: correction between HLA-E gene nucleotide-26 with age among healthy blood donors. [file 9847708.f1.docx]

**Supplement Table 1: Hardy Weinberg Equilibrium test**

| Group | Healthy blood donors | Leukemia patients | Expect value | P value |
| --- | --- | --- | --- | --- |
| TT | 1 | 0 | 1.98 | >0.05 |
| GT | 40 | 19 | 56.05 |  |
| GG | 185 | 209 | 395.96 |  |

**Supplement Table 2: Correction between HLA-E gene nucleotide-26 with gender among healthy blood donors**

| Group | -26G | -26T | P value |
| --- | --- | --- | --- |
| Male | 205 | 21 | 0.870 |
| Female | 206 | 20 |  |

**Supplement Table 3: Correction between HLA-E gene nucleotide-26 with age among healthy blood donors**

| Group | -26T | -26G | P value |
| --- | --- | --- | --- |
| 18-29 | 10 | 96 | 0.866 |
| 30-39 | 7 | 93 |  |
| 40-49 | 15 | 173 |  |
| 50-65 | 6 | 52 |  |
